# Blood Pressure Control in Individuals With Hypertension Who Used a Digital, Personalized Nutrition Platform: Longitudinal Study

**DOI:** 10.2196/35503

**Published:** 2022-03-17

**Authors:** Shivani Bakre, Benjamin Shea, Jason Langheier, Emily A Hu

**Affiliations:** 1 Foodsmart San Francisco, CA United States; 2 Department of Epidemiology Johns Hopkins Bloomberg School of Public Health Baltimore, MD United States; 3 Department of Biostatistics Harvard T H Chan School of Public Health Boston, MA United States

**Keywords:** blood pressure, hypertension, systolic, diastolic, digital, nutrition, meal planning, food environment, food ordering, food purchasing, cardiology, digital health, digital platform, health technology, platform usability

## Abstract

**Background:**

While there is a strong association between adhering to a healthy dietary pattern and reductions in blood pressure, adherence remains low. New technologies aimed to help facilitate behavior change may have an effect on reducing blood pressure among individuals with hypertension.

**Objective:**

This study aims to evaluate characteristics of participants with stage 2 hypertension who used Foodsmart and to assess changes in systolic blood pressure (SBP) and diastolic blood pressure (DBP).

**Methods:**

We analyzed demographic, dietary, and clinical characteristics collected from 11,934 adults with at least two blood pressure readings who used the Foodsmart platform. Stage 2 hypertension was defined as SBP ≥140 mmHg or DBP ≥90 mmHg. We calculated mean changes in blood pressure among participants with stage 2 hypertension and stratified by length of follow-up and the covariates associated with achieving blood pressure levels below stage 2 hypertension. We compared changes in diet quality and weight between participants with stage 2 hypertension at baseline who achieved stage 1 hypertension or below and those who did not.

**Results:**

We found that 10.63% (1269/11,934) of participants had stage 2 hypertension at baseline. Among Foodsmart participants with stage 2 hypertension at baseline, SBP and DBP decreased, on average, by 5.7 and 4.0 mmHg, respectively; 33.02% (419/1269) of participants with stage 2 hypertension at baseline achieved blood pressure levels below stage 2 hypertension (SBP <140 mmHg and DBP <90 mmHg). Using a multivariable ordinal logistic regression model, changes in Nutriscore (*P*=.001) and weight (*P*=.04) were statistically significantly associated with changes in blood pressure categories for users with stage 2 hypertension at baseline. Using a multivariable logistic regression model, we found that baseline Nutriscore, change in Nutriscore, and change in weight were associated with greater likelihood of users with stage 2 hypertension at baseline achieving a lower blood pressure category.

**Conclusions:**

This study evaluated changes in SBP and DBP among users (with hypertension) of the Foodsmart platform and found that those with stage 2 hypertension, on average, improved their blood pressure levels over time.

## Introduction

Almost half of all adults living in the United States have hypertension, with only 1 in 4 having their condition under control [1]. Hypertension occurs when the body’s blood vessels narrow, which results in blood exerting a greater pressure on the walls of one’s blood vessels and the heart working harder [2]. Uncontrolled hypertension can also lead to a myriad of health complications as it can result in narrow, weak, or thick blood vessels, which prevent organs from functioning normally. Notably, it can cause aneurysm, heart disease, or stroke, with heart disease and stroke being the leading causes of death in the United States [1,3]. Consequently, this condition costs the US health care system about US $131 billion annually [4].

A healthy diet is an important part of an individual’s toolkit to reduce their blood pressure. Previous studies have shown that a healthy dietary pattern can reduce hypertension to controlled levels. For example, the Dietary Approaches to Stop Hypertension (DASH) diet is composed of fruits, vegetables, and low-fat dairy products. Filippou et al [5] found in a systematic review of studies that the DASH diet resulted in significant reductions in blood pressure both for those with hypertension and those without. After such success in these trials, DASH is now recommended by the National Institutes of Health (NIH) to reduce hypertension [6]. Despite robust evidence supporting the relationship between healthy eating and lower risk of hypertension, barriers related to time, finances, information, and accessibility in obtaining healthy foods prevent people from starting and sustaining such changes over their lifetime.

Foodsmart, a digital nutrition platform, is a potential solution to address these barriers. The platform helps its users improve their health by providing them information about their current diet and pinpointing areas of improvement, while also providing personalized recipes and meal planning options to achieve target goals. The platform also assists in purchasing healthier foods with direct integration from meal plans to grocery lists to retailers through an ad-free environment. Consequently, Foodsmart is able to create sustainable behavior change for its users by making the process easy, quick, and affordable. Previous research has shown that this sustained behavior change has resulted in sustained changes in biometrics as well. Hu et al [7] found that Foodsmart members with obesity have been able to achieve sustained weight loss over the time during which they have used the platform.

Previous studies have also shown the effectiveness of digital nutrition interventions in helping participants lower their blood pressure. Milani et al [8] provided a digital intervention that consisted of lifestyle recommendations as well as medication management to assist patients in reducing their blood pressure. Participants were asked to measure their blood pressure at least once a week on purchased blood pressure devices. They found that in the digital intervention groups patients saw an average decrease in blood pressure of 14 mmHg in systolic blood pressure (SBP) and 5 mmHg in diastolic blood pressure (DBP) after 90 days compared with those on usual care with a reduction of 4 mmHg/2 mmHg [8]. This study displays what is possible at the higher end of blood pressure reductions over 90 days given that this was a partially medication-guided intervention. Steinberg et al [9] implemented a digital intervention to attempt to increase DASH adherence and thus lower blood pressure more through the use of a mobile diet-tracking app. After 3 months, participants had an average reduction of 2.8 mmHg of SBP and 3.6 mmHg for DBP [9]. Foodsmart, however, does not provide medication management services and differs from the latter intervention in its digital interface, personalized meal planning, and online food ordering system. Therefore, Foodsmart is well-positioned to help its participants with hypertension make sustainable, holistic changes in their health by both providing them with nutritional information and adjusting their food purchasing environment to facilitate behavior change.

This study aims to characterize Foodsmart participants with stage 2 hypertension, as well as identify what characteristics are associated with returning to stage 1 hypertension levels or lower among those with stage 2 hypertension. It also aims to evaluate changes in blood pressure levels and other biometrics over time among participants with hypertension.

## Methods

### Study Sample

As of August 2021, 106,816 participants of Foodsmart who were older than 18 years living the United States and enrolled since January 2016 had entered plausible systolic and diastolic readings (SBP between 80 and 300 mmHg, exclusive; DBP between 40 and 200 mmHg, exclusive). Of those Foodsmart participants, 11,934 had reported at least two systolic/diastolic readings, with the first and last reported readings at least 30 days apart. Our final sample size was 11,934 participants with at least two reports of SBP and DBP.

### Foodsmart

Foodsmart is a digital nutrition platform that facilitates sustained dietary and behavior change through 2 main components, FoodSmart and FoodsMart. Foodsmart has been described previously [10]. In brief, participants on Foodsmart complete an online dietary assessment, Nutriquiz, to evaluate current diet quality. Based on the results of the Nutriquiz, personalized meal plans, aligned with their dietary preferences, are created that are converted into grocery lists and integrated into online order and delivery of meal kits, prepared foods, and groceries.

Foodsmart is available through health plans and employers and can be accessed via the web or the iOS or Android operating system.

### Measurements of SBP, DBP, and Weight

Foodsmart participants were able to input their biometrics such as blood pressure, height, and weight, and were able to report new biometrics at any time. Because of potential errors in participants self-reporting their biometrics, the following values were considered as outliers and removed: SBP ≤80 mmHg or ≥300 mmHg, DBP ≤40 mmHg or ≥200 mmHg, weight ≤27.2 kg or ≥181.1 kg, and BMI ≤15 kg/m^2^ or ≥50 kg/m^2^. Length of follow-up was calculated as the number of days between the first and last systolic/diastolic measures inputted. We defined normotension as SBP <120 mmHg and DBP <80 mmHg, elevated blood pressure as SBP between 120 and 129 mmHg (inclusive) and DBP <80, stage 1 hypertension as either SBP between 130 and 139 mmHg (inclusive) or DBP between 80-89 mmHg (inclusive), and stage 2 hypertension as either SBP ≥140 mmHg or DBP ≥90 mmHg, as defined by the American Heart Association [11]. These rules were applied to both the first and last systolic/diastolic measures reported. We used the stage 2 hypertension cutoff for the primary analysis, and the stage 1 hypertension cutoff for the sensitivity analysis. Changes in blood pressure were calculated by subtracting the end SBP and DBP readings from the baseline SBP and DBP readings, respectively. Percent changes were calculated for both SBP and DBP readings by dividing the change in SBP/DBP readings by the baseline SBP/DBP measurements.

Baseline BMI was calculated as the baseline weight entered in kilograms divided by height in meters squared (kg/m^2^). Participants’ baseline BMI was categorized as normal BMI (BMI <25 kg/m^2^), overweight (25-29.9 kg/m^2^), or obese (BMI ≥30 kg/m^2^). Participants were also able to report any conditions they currently had (eg, diabetes, high blood pressure) in the Nutriquiz.

### Dietary Assessment

Participants self-reported their food intake and dietary habits in the Foodsmart platform. Participants were asked to fill out a 53-item food frequency questionnaire called Nutriquiz (adapted from the National Cancer Institute Diet History Questionnaire I) when they sign up [12]. Demographic information (age, sex, height), weight, and daily dietary intake (added sugars, fiber, fruits, vegetables, whole grains, fats, proteins, water, and sodium) were collected in the Nutriquiz.

Based on responses from the Nutriquiz and the daily dietary intake, a score (Nutriscore) from 0 to 70 was calculated to assess overall diet quality, with 70 being the highest diet quality. The Nutriscore is based on the Alternative Healthy Eating Index-2010 and the Commonwealth Scientific and Industrial Research Organization Healthy Diet Score [13,14]. Change in the Nutriscore was calculated as a participant’s last Nutriscore minus the first Nutriscore. A positive change in Nutriscore indicates the participant improved their nutrition.

### Statistical Analysis

We performed exploratory data analyses to assess the baseline demographic characteristics, SBP and DBP levels, and diet quality of the total study population, stratified by whether participants had stage 2 hypertension at baseline or not. We reported categorical variables as number of participants (percentage of study population) and continuous variables as mean (SD). We used chi-square tests to assess whether categorical variables are independent of baseline stage 2 hypertension status, and 2-sample *t* tests (unpaired) to evaluate differences in continuous variables.

Univariate and multivariable ordinal logistic regression analyses were used to estimate the odds ratios (ORs) and 95% CIs of achieving different blood pressure categories at different thresholds (eg, using the elevated blood pressure category as the threshold, the odds of achieving below stage 1 hypertension and stage 2 hypertension). Four blood pressure categories were considered: normotension, elevated blood pressure, stage 1 hypertension, and stage 2 hypertension. Univariate and multivariable logistic regression analyses were used to estimate the ORs and 95% CIs of achieving blood pressure levels below stage 2 hypertension. The multivariable logistic regression and multivariable ordinal logistic regression models were both mutually adjusted for sex, age category, baseline BMI category, baseline Nutriscore, change in Nutriscore, change in weight, baseline SBP, baseline DBP, diabetes, and high cholesterol.

Among participants who had stage 2 hypertension, we calculated the mean changes in SBP and DBP overall, and by time of follow-up at different thresholds (≥6, ≥12, and ≥24 months). We used paired *t* tests to evaluate whether the changes in SBP and DBP were statistically significant. In addition, we calculated the mean percentage change for SBP and DBP. In a sensitivity analysis, we used a threshold of stage 1 hypertension or higher (SBP ≥130 mmHg or DBP ≥80 mmHg) to calculate mean changes in HbA1c.

We also calculated the percentage of participants with stage 2 hypertension at baseline who achieved stage 1 hypertension or lower by the end of follow-up, and stratified by follow-up length.

To further explore the impact on SBP and DBP, we examined changes in weight, diet quality, SBP, and DBP, stratified by whether participants with stage 2 hypertension at baseline achieved stage 1 hypertension or lower (SBP <130 mmHg and DBP <80 mmHg) by the end of follow-up.

We considered *P* values less than .05 to be significant for all tests. R Studio version 1.4.1106 and R version 4.0.5 (R Foundation) were used for all analyses.

### Ethical Approval

The study was declared exempt from institutional review board oversight by the Pearl Institutional Review Board given the retrospective design of the study and the less than minimal risk to participants.

## Results

### Participant Characteristics

Baseline demographics and biometrics of the total study sample stratified by a threshold of stage 2 hypertension status are shown in Table 1. Of participants with at least two blood pressure entries, 47.15% (5627/11,934) were classified as having stage 1 hypertension or higher at baseline, and 10.63% (1269/11,934) were classified as having stage 2 hypertension at baseline.

There were 11,934 participants included in the total study sample; 69.93% (8346/11,934) of the participants were female, 19.44% (2320/11,934) were 60 years or older, 68.46% (8170/11,934) were either overweight or obese, 17.80% (2124/11,934) reported having diabetes, and 37.91% (4254/11,934) reported having high cholesterol. The mean weight was 83.9 (SD 22.1) kg, the mean baseline Nutriscore was 33.8 (SD 8.5) points, and the mean change in the Nutriscore was 1.7 (SD 7.1) points. The mean follow-up length was 8.8 (SD 9.5) months and ranged from 1 to 65 months. Compared with participants who did not have stage 2 hypertension, participants who did have stage 2 hypertension were more likely to be male, to have a higher weight and BMI, to have a lower baseline Nutriscore, to have a longer length of follow-up, and to self-report having diabetes or high cholesterol. Participants with stage 2 hypertension at baseline were also more likely to have a higher increase in Nutriscore compared with participants without stage 2 hypertension at baseline, although the difference was not statistically significant (*P*=.28).

To better understand what blood pressure category participants attained at the end of follow-up, we used ordinal logistic regression to examine the association of covariates with the end blood pressure categories among participants who had stage 2 hypertension at baseline (Table 2). Threshold 1 indicates the probability of stage 2 hypertension, stage 1 hypertension, or elevated blood pressure versus normotension. Threshold 2 indicates the probability of stage 2 hypertension or stage 1 hypertension versus elevated blood pressure or normotension. Threshold 3 indicates the probability of stage 2 hypertension versus stage 1 hypertension, elevated blood pressure, or normotension. In the univariate ordinal logistic regression models, participants who were female were less likely to end in the elevated blood pressure or higher categories versus the normotensive blood pressure category on average (OR 0.75, 95% CI 0.59-0.96; *P*=.02). Participants with a higher baseline Nutriscore were less likely to end in the elevated blood pressure or higher categories versus the normotensive blood pressure category on average (OR 0.97, 95% CI 0.94-0.99; *P*=.01). Participants whose Nutriscore increased at the end of follow-up were less likely to end in the elevated blood pressure or higher categories versus the normotensive category (OR 0.96, 95% CI 0.93-0.99; *P*=.008). Participants who gained more weight at the end of follow-up were more likely to end in the elevated blood pressure or higher categories versus the normotensive category on average (OR 1.02, 95% CI 1.01-1.04; *P*=.004).

**Table 1:** Baseline characteristics of total study sample and by baseline hypertension status.

Characteristic			All participants (n=11,934)		Below stage 2 hypertension (n=10,665)		Stage 2 hypertension (n=1269)		*P* value^a^	
Female, n (%)			8346 (69.93)		7539 (70.69)		807 (63.59)		<.001	
**Age (years), n (%)**									<.001	
	<40	2929 (24.54)		2697 (25.29)		232 (18.28)				
	40-59	6685 (56.02)		5927 (55.57)		758 (59.73)				
	≥60s	2320 (19.44)		2041 (19.14)		279 (21.99)				
Weight (kg), n; mean (SD)			11,919; 83.9 (22.1)		10,656; 82.6 (21.6)		1263; 93.0 (24.2)		<.001	
Change in weight (kg), n; mean (SD)			7934; –0.4 (7.5)		7111; –0.4 (7.3)		726; –0.7 (8.7)		<.001	
**BMI category, n (%)**									<.001	
	Normal	3621 (30.34)		3418 (32.05)		203 (16.00)				
	Overweight	3691 (30.93)		3339 (31.31)		352 (27.74)				
	Obese	4479 (37.53)		3803 (35.66)		676 (53.27)				
	Missing	143 (1.20)		105 (0.98)		38 (2.99)				
Baseline systolic blood pressure (mmHg), n; mean (SD)			11,934; 120 (13.0)		10,665; 118 (9.8)		1269; 140 (19.1)		<.001	
Baseline diastolic blood pressure (mmHg), n; mean (SD)			11,934; 76.0 (9.6)		10,665; 74.4 (7.6)		1269; 89.5 (13.4)		<.001	
Follow-up duration (months), n; mean (SD)			11,934; 8.8 (9.5)		10,665; 8.7 (9.3)		1269; 9.8 (10.9)		.03	
Diabetes, n (%)			2124 (17.80)		1787 (16.76)		337 (26.56)		<.001	
High cholesterol, n (%)			4254 (35.65)		3701 (34.70)		553 (43.58)		<.001	
Baseline Nutriscore (0-70), n; mean (SD)			11,934; 33.8 (8.5)		10,665; 34.2 (8.5)		1269; 31.3 (8.5)		<.001	
Change in Nutriscore, n; mean (SD)			11,551; 1.7 (7.1)		10,335; 1.6 (7.1)		1196; 1.9 (7.3)		.28	

^a^Chi-square tests and 2-sample *t* tests (paired) were used to test differences for categorical and continuous variables, respectively.

**Table 2:** Association between predictors and likelihood of blood pressure categories at the end of follow-up in the univariate and multivariable ordinal logistic regression models.

Association			Univariate odds ratio (95% CI)		*P* value		Multivariable odds ratio (95% CI)		*P* value
Sex (female)			0.75 (0.59-0.96)		.02		0.82 (0.60-1.12)		.22
**Age**									
	<40	1 (reference)				1 (reference)			
	40-59	0.76 (0.55-1.05)		.09		0.79 (0.53-1.18)		.24	
	≥60	0.61 (0.42-0.88)		.009		0.68 (0.42-1.09)		.11	
Baseline Nutriscore (0-70; per 2 points)			0.97 (0.94-0.99)		.01		0.96 (0.92-0.99)		.02
Change in Nutriscore (per 2 points)			0.96 (0.93-0.99)		.008		0.93 (0.89-0.97)		.001
**Baseline BMI category**									
	Normal	1 (reference)				1 (reference)			
	Overweight	0.96 (0.66-1.37)		.81		0.90 (0.56-1.45)		.67	
	Obese	0.93 (0.67-1.30)		.69		0.78 (0.50-1.23)		.29	
Change in weight (kg)			1.02 (1.01-1.04)		.004		1.02 (1.00-1.04)		.04
Length of follow-up (per 6 months)			0.91 (0.86-0.97)		.002		0.97 (0.90-1.04)		.40
Diabetes			0.91 (0.71-1.18)		.50		1.14 (0.81-1.59)		.46
High cholesterol			0.84 (0.66-1.05)		.13		0.80 (0.59-1.08)		.15
Baseline systolic (per 5 mmHg)			0.98 (0.95-1.01)		.17		1.00 (0.96-1.05)		.96
Baseline diastolic (per 5 mmHg)			1.04 (1.00-1.09)		.07		1.05 (0.99-1.12)		.12

After adjusting for all other covariates in the multivariable ordinal logistic regression model, change in Nutriscore was inversely associated with ending with elevated blood pressure, stage 1 hypertension, or stage 2 hypertension (OR 0.93, 95% CI 0.89-0.97; *P*=.001); for every 2-point increase in Nutriscore, a participant was 7% less likely to end in the elevated blood pressure or higher categories versus the normotensive category on average. Change in weight was positively associated with ending in the elevated blood pressure or higher categories (OR 1.02, 95% CI 1.00-1.04; *P*=.04). For every 1-kg increase in weight, a participant was 2% more likely to end with elevated blood pressure or higher versus normotension, on average.

We further examined the association between baseline characteristics and odds of achieving a blood pressure category below stage 2 hypertension in the univariate and multivariable logistic regression models (Table 3). In the univariate regression models, participants who were female were 30% more likely to achieve an end blood pressure category below stage 2 hypertension than participants who were male (OR 1.30, 95% CI 1.02-1.67; *P*=.04). Participants classified in the age group 60 years and older were 68% more likely to achieve an end blood pressure category below stage 2 hypertension than participants classified in the less than 40-year-old group (OR 1.68, 95% CI 1.15-2.46; *P*=.007). Participants with a higher baseline Nutriscore were more likely to achieve an end blood pressure category below stage 2 hypertension (OR 1.04, 95% CI 1.01-1.06; *P*=.01). Participants with an increase in Nutriscore were more likely to achieve an end blood pressure category below stage 2 hypertension (OR 1.04, 95% CI 1.01-1.08; *P*=.01). Participants with a decrease in weight were more likely to achieve an end blood pressure category below stage 2 hypertension (OR 0.98, 95% CI 0.96-0.99; *P*=.003). Participants with a longer length of follow-up were more likely to achieve an end blood pressure category below stage 2 hypertension (OR 1.11, 95% CI 1.05-1.19; *P*=.001).

**Table 3:** Odds of achieving blood pressure below stage 2 hypertension for users with stage 2 hypertension at baseline in univariate and multivariable logistic regression analyses.

Variables			Univariate odds ratio (95% CI)	*P* value	Multivariable odds ratio (95%)	*P* value
Sex (female)			1.30 (1.02-1.67)	.04	1.16 (0.85-1.61)	.36
**Age (years)**						
		<40	1 (reference)		1 (reference)	
		40-59	1.36 (0.98-1.89)	.07	1.34 (0.89-2.03)	.17
		≥60	1.68 (1.15-2.46)	.007	1.58 (0.96-2.61)	.07
Baseline Nutriscore (0-70; per 2 points)			1.04 (1.01-1.06)	.01	1.05 (1.01-1.10)	.01
Change in Nutriscore (per 2 points)			1.04 (1.01-1.08)	.01	1.07 (1.03-1.12)	.003
**Baseline BMI category**						
	Normal		1 (reference)		1 (reference)	
	Overweight		1.14 (0.79-1.67)	.48	1.21 (0.74-2.00)	.45
	Obese		1.19 (0.85-1.68)	.31	1.43 (0.89-2.31)	.14
Change in weight (kg)			0.98 (0.96-0.99)	.003	0.98 (0.96-1.00)	.03
Length of follow-up (per 6 months)			1.11 (1.05-1.19)	.001	1.04 (0.97-1.13)	.26
Diabetes			1.15 (0.88-1.49)	.30	0.92 (0.64-1.30)	.62
High cholesterol			1.21 (0.96-1.54)	.11	1.23 (0.90-1.68)	.20
Baseline systolic (per 5 mmHg)			1.02 (0.99-1.06)	.14	1.01 (0.96-1.05)	.78
Baseline diastolic (per 5 mmHg)			0.96 (0.92-1.01)	.12	0.96 (0.90-1.02)	.23

After adjusting for all other covariates in the multivariable logistic regression model, we found that for every 2-point increase in baseline Nutriscore, a user would be 5% more likely to achieve an end blood pressure category below stage 2 hypertension (OR 1.05, 95% CI 1.01-1.10; *P*=.01). In addition, adjusting for all other covariates, for every 2-point increase in change in Nutriscore, a participant would be 7% more likely to achieve an end blood pressure category below stage 2 hypertension (OR 1.07, 95% CI 1.03-1.12; *P*=.003). Adjusting for all other covariates, for every 1-kg increase in change in weight, a participant would be 2% less likely to achieve an end blood pressure category below stage 2 hypertension (OR 0.98, 95% CI 0.96-1.00; *P*=.03).

### Changes in SBP and DBP

Multimedia Appendices 1 and 2 show the mean and percent change in SBP and DBP, respectively, for participants who had stage 2 hypertension at baseline. Changes were calculated overall and by length of follow-up, at ≥6, ≥12, and ≥24 months. The mean changes in SBP for overall and subsetting to length of follow-up ≥6, ≥12, and ≥24 months were –5.7 (SD 14.6), –7.0 (SD 15.7), –8.0 (SD 14.3), and –7.4 (SD 13.3) mmHg, respectively. The mean percentage changes in SBP were –3.5% (SD 10.3%), –4.4% (SD 10.4%), –5.2% (SD 9.4%), and –4.8% (SD 8.6%) for overall, ≥6, ≥12, and ≥24 months, respectively. The mean changes in DBP for overall and subsetting to length of follow-up ≥6, ≥12, and ≥24 months were –4.0 (SD 10.6), –4.4 (SD 10.5), –5.0 (SD 9.8), and –4.3 (SD 10.1) mmHg, respectively. The mean percentage changes in DBP were –3.8% (SD 9.7%), –4.4% (SD 9.7%), –5.1% (SD 8.9%), and –4.3% (SD 8.9%) for overall, ≥6, ≥12, and ≥24 months, respectively. Using paired *t* tests, all changes between baseline and end SBP and DBP readings were statistically significant at the *P*<.05 level. Systolic and diastolic BP for overall, ≥6, ≥12, and ≥24 months were all *P*<.001.

For participants who had stage 1 hypertension at baseline, the mean changes in SBP for overall and subsetting to length of follow-up ≥6, ≥12, and ≥24 months were –2.0 (SD 9.4), –2.4 (SD 10.1), –3.0 (SD 10.3), and –2.7 (SD 9.9) mmHg, respectively. Mean percentage changes in SBP were –1.3% (SD 6.9%), –1.6% (SD 7.2%), –2.0% (SD 7.4%), and –1.8% (SD 7.1%) for overall, ≥6, ≥12, and ≥24 months, respectively. The mean changes in DBP for overall and subsetting to length of follow-up ≥6, ≥12, and ≥24 months were –1.8 (SD 6.9), –2.1 (SD 6.9), –2.5 (SD 7.3), and –2.4 (SD 7.1) mmHg, respectively. The mean percentage changes in DBP were –1.9% (SD 7.4%), –2.3% (SD 7.5%), –2.7% (SD 8.1%), and –2.6% (SD 7.6%) for overall, ≥6, ≥12, and ≥24 months, respectively. Using paired *t* tests, all changes between baseline and end SBP and DBP readings were statistically significant at the *P*<.05 level. Systolic and diastolic BP for overall, ≥6, ≥12, and ≥24 months were all *P*<.001.

We evaluated the percentage of participants who had stage 2 hypertension at baseline who achieved blood pressure levels below stage 2 hypertension, systolic reading <140 mmHg and diastolic reading <90 mmHg, at the end of follow-up (Multimedia Appendix 3). Across all length-of follow-up periods, 33.02% (419/1269) of participants with stage 2 hypertension at baseline lowered their blood pressure to below stage 2 hypertension by the end of follow-up. Among participants whose follow-up time was longer than 6, 12, and 24 months, 37.6% (238/633), 42.5% (131/308), and 38.4% (66/172) of participants achieved blood pressure levels below stage 2 hypertension by the end of follow-up.

We examined the change in weight and Nutriscore for participants who lowered their blood pressure levels to below stage 2 hypertension versus those who remained in the stage 2 hypertension category (Multimedia Appendix 4). Participants who changed from stage 2 hypertension to below stage 2 hypertension lost 1.8 more kilograms in weight (0.0 kg versus 1.8 kg lost) and had 1.2 points higher (1.5-point increase versus 2.7-point increase) in their diet quality (Nutriscore) than those who remained in the stage 2 hypertension category.

## Discussion

In this study, we found that 10.63% (1269/11,934) of Foodsmart participants had stage 2 hypertension at baseline. Foodsmart participants with stage 2 hypertension at baseline were more likely to be male, older, have a higher weight and BMI, have additional comorbidities (diabetes, high cholesterol), have a lower Nutriscore, and have a longer follow-up. For participants with stage 2 hypertension, blood pressure reduced by 5.7 mmHg/4.0 mmHg on average over a follow-up of 9.8 (SD 10.9) months. Among participants with stage 2 hypertension at baseline, 33.02% (419/1269) lowered their blood pressure to stage 1 hypertension or below. Furthermore, there were greater reductions in weight changes and increases in Nutriscores among those with stage 2 hypertension at baseline who achieved blood pressure levels below stage 2 hypertension. Our findings suggest that participant use of Foodsmart may be associated with reductions in blood pressure for patients with stage 2 hypertension.

Previous research confirms several of our findings. Through Korea’s National Health and Nutrition Examination Survey, Noh et al [15] confirmed that, in line with our baseline analysis, those with hypertension are more likely to have additional comorbidities, noting that these comorbidities (obesity, dyslipidemia, and impaired fasting glucose) are also likely to be associated with age and male sex. Additional studies have provided further context to these findings. Weight, in particular, has been long-established to increase the risk of hypertension [16]. Having a BMI greater than or equal to 30 kg/m^2^ (categorized as obese) has been associated with a 3.5 times higher likelihood of developing hypertension. In addition, previous research has shown that diabetes and hypertension often occur together because they are mediated by similar physiologic conditions, such as impaired circadian blood pressure rhythm, renin-angiotensin-aldosterone blood pressure system dysregulation, microvascular and macrovascular damage, and more [17,18]. Finally, prior research also suggests that dyslipidemia may be a risk factor for hypertension [19]. Dyslipidemia is known to cause endothelial damage, which results in the constriction of cardiovascular vessels as opposed to their dilation. This damage may consequently result in hypertension. In line with our findings, Neter et al [20] found a dose-response relationship between weight change and blood pressure reduction. They found a reduction in blood pressure of −1.05 mmHg/−0.92 mmHg per kilogram of weight loss. In addition, a meta-analysis on the DASH diet found that improved nutrition is associated with greater decreases in blood pressure, with the mean difference of SBP and DBP being –3.2 and –2.5 mmHg, respectively, from a systematic review of 30 randomized clinical trials [5].

Individuals with hypertension are estimated to incur US $1920 higher incremental health care expenditures and nearly triple the medication expenditures compared with individuals without hypertension [4]. Drugs such as angiotensin-converting enzyme (ACE) inhibitors and calcium channel blockers account for a large proportion of these expenditures. Given the high cost of medications, prevention and management of hypertension through healthier eating could supplement hypertension drugs and potentially lower its usage. Unfortunately, we do not know whether participants were on blood pressure medications before or during enrollment on the Foodsmart platform. Despite this, we can estimate the difference in costs between prescription medications and Foodsmart. As of 2021, the Foodsmart platform on average costs US $12.30 per eligible member annually. Using the results above, a 5-mmHg reduction in SBP and DBP would cost US $12.30 on average for participants on the Foodsmart platform for at least a year. Using lisinopril (ACE inhibitor), by contrast, would cost US $48 on average to reduce SBP/DBP by 21.4/15.7 mmHg [21]. The Foodsmart platform could be a cost-effective program to complement the use of standard hypertension medications.

There are some important limitations to note for this study. The first is that blood pressure values were self-reported and not clinically validated. Given that only blood pressure values were reported, we do not have information on which blood pressure devices were used or if standard protocol, such as the average of 3 consecutive measurements, was used. We assumed participants inputted their last recorded blood pressure values from a clinical setting. Nevertheless, we have reason to believe these values should still be approximately accurate, because blood pressure is not a required input for the app, and therefore those who did input blood pressure were most likely motivated by personal tracking. In addition, because values were self-reported, follow-up time was based on when the biometrics were entered as opposed to when blood pressure was measured. Therefore, the first measurements of SBP and DBP recorded may not necessarily align with the beginning of Foodsmart enrollment. Another possible issue is selection bias for individuals with hypertension who use Foodsmart. Those with hypertension who use the app may be more likely to make lifestyle changes because they are using the app for the purposes of improving their nutrition. These users may also be working to make lifestyle changes outside of the app that affect their blood pressure. Therefore, we cannot state that Foodsmart caused these changes in blood pressure. To make statements about causation, a randomized controlled trial must be conducted. Next, we were not able to collect data on other factors that might affect hypertension status. For instance, participants’ personal or family medical histories may have an influence on hypertension status. We lack information on whether participants were taking blood pressure medications before or during usage of the Foodsmart platform or if participants changed blood pressure medication during enrollment. Finally, we did not have data on socioeconomic factors, such as education, which may confound the associations. Further research is necessary to understand these additional variables. We also did not examine the frequency of Foodsmart app use, which may affect the associations found in our analyses.

Our analysis also has many strengths. This is the first study to examine the real-life impact of behavioral change as a result of online food ordering, dietary education, and meal planning through a digital intervention and its impact on hypertension. Through analyzing data from Foodsmart’s large user base, we were able to gather information about real-world associations between changes in diet and blood pressure levels. Furthermore, Foodsmart participants had a wide range of enrollment lengths, which allowed us to evaluate changes in blood pressure over different lengths of time, including follow-up time of more than 2 years.

In summary, this study assessed changes in self-reported SBP and DBP for participants using a digital nutrition platform that offers personalized meal planning, food ordering, grocery discounts, and price comparisons. Further research through a randomized clinical trial is warranted to evaluate the causal effect between the use of the Foodsmart platform and changes in blood pressure among participants.

## Appendix

Multimedia Appendix 1 Mean change in SBP among participants who had stage 2 hypertension at baseline.

Multimedia Appendix 2 Mean change in DBP among participants who had stage 2 hypertension at baseline.

Multimedia Appendix 3 Percent of users who changed from stage 2 hypertension at baseline to below stage 2 hypertension at the end of follow-up.

Multimedia Appendix 4 Change in biometrics stratified by whether participants with stage 2 hypertension at baseline achieved a blood pressure below stage 2 hypertension.

## Abbreviations

ACE: angiotensin-converting enzyme
DASH: Dietary Approaches to Stop Hypertension
DBP: diastolic blood pressure
NIH: National Institutes of Health
OR: odds ratio
SBP: systolic blood pressure
