# Supplementary figures and images for: Blood Pressure Control in Individuals With Hypertension Who Used a Digital, Personalized Nutrition Platform: Longitudinal Study
Source: JMIR Form Res. 2022 Mar 17;6(3):e35503. doi: 10.2196/35503 (PMC8972110; doi:10.2196/35503)

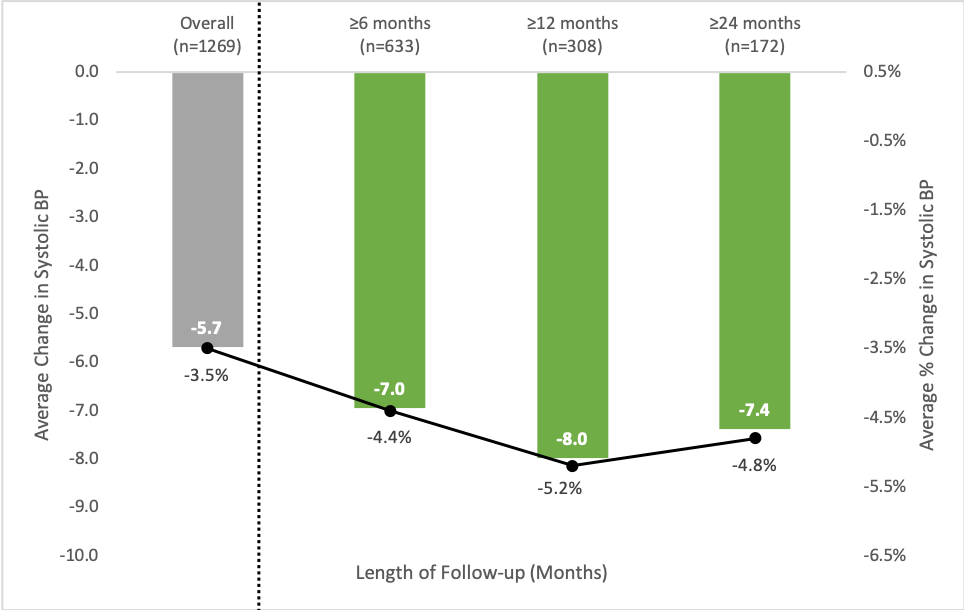

Supplement: Multimedia Appendix 1 [file formative_v6i3e35503_app1.png]

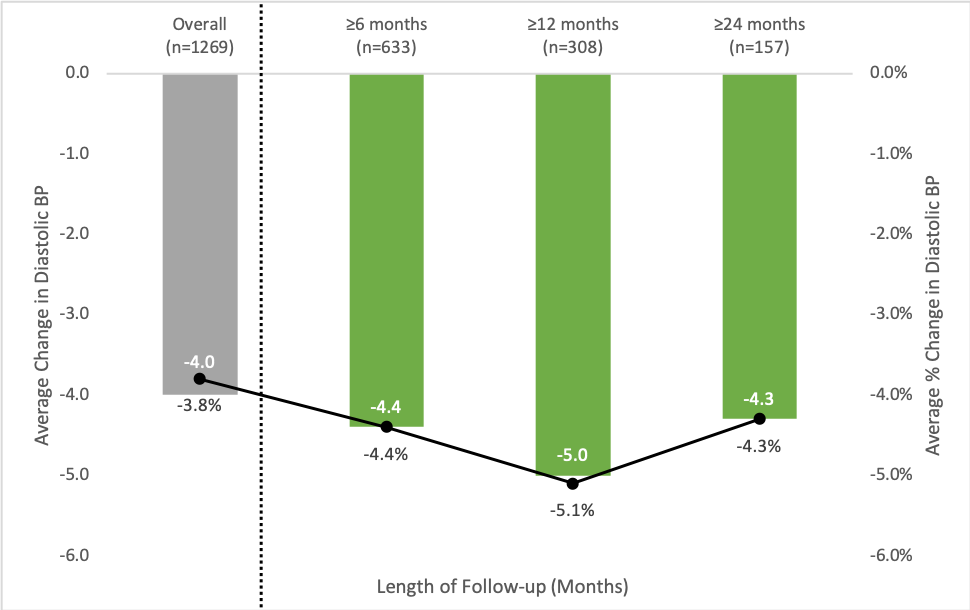

Supplement: Multimedia Appendix 2 [file formative_v6i3e35503_app2.png]

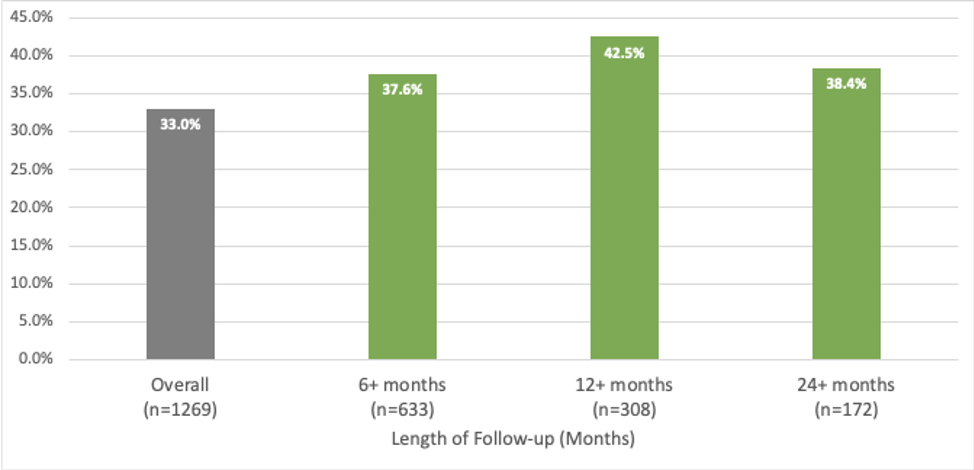

Supplement: Multimedia Appendix 3 [file formative_v6i3e35503_app3.png]

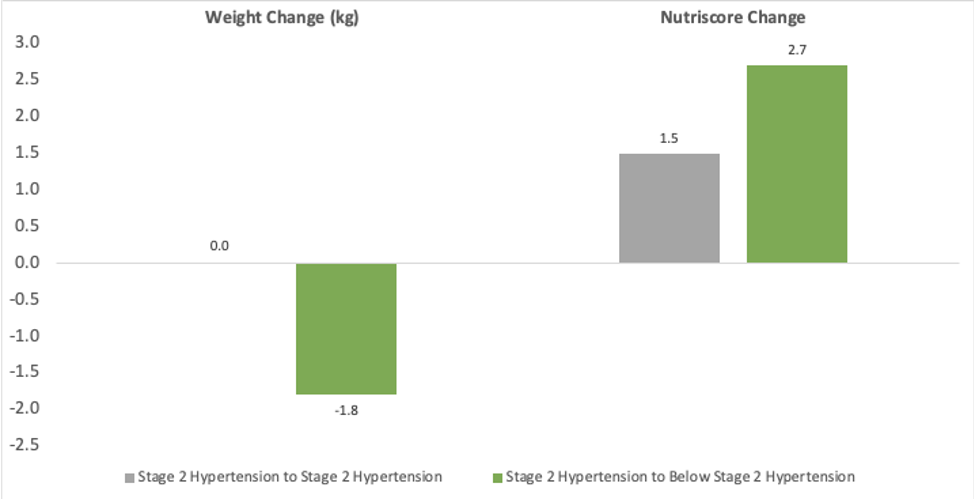

Supplement: Multimedia Appendix 4 [file formative_v6i3e35503_app4.png]
